# Improving the coverage of the PMTCT programme through a participatory quality improvement intervention in South Africa

**DOI:** 10.1186/1471-2458-9-406

**Published:** 2009-11-05

**Authors:** Tanya Doherty, Mickey Chopra, Duduzile Nsibande, Dudu Mngoma

**Affiliations:** 1 Health Systems Research Unit, Medical Research Council, Francie van Zyl Drive, Tygerberg, South Africa; 2 Health Systems Research Unit, Medical Research Council, 491 Ridge Road, Durban, South Africa; 3 School of Public Health, University of the Western Cape, Modderdam Road, Bellville, South Africa

## Abstract

**Background:**

Despite several years of implementation, prevention of mother-to-child transmission (PMTCT) programmes in many resource poor settings are failing to reach the majority of HIV positive women. We report on a data driven participatory quality improvement intervention implemented in a high HIV prevalence district in South Africa.

**Methods:**

A participatory quality improvement intervention was implemented consisting of an initial assessment undertaken by a team of district supervisors, workshops to assess results, identify weaknesses and set improvement targets and continuous monitoring to support changes.

**Results:**

The assessment highlighted weaknesses in training and supervision. Routine data revealed poor coverage of all programme indicators except HIV testing. Monthly support to all facilities took place including an orientation to the PMTCT protocol, review of local data and identification of bottlenecks to optimal coverage using a continuous quality improvement approach. One year following the intervention large improvements in programme indicators were observed. Coverage of CD4 testing increased from 40 to 97%, uptake of maternal nevirapine from 57 to 96%, uptake of infant nevirapine from 15 to 68% and six week PCR testing from 24 to 68%.

**Conclusion:**

It is estimated that these improvements in coverage could avert 580 new infant infections per year in this district. This relatively simple participatory assessment and intervention process has enabled programme managers to use a data driven approach to improve the coverage of this important programme.

## Background

UNAIDS estimates that approximately 370 000 children were infected with HIV in 2007[1]. More than 90% of these infections were caused by vertical transmission from mother to infant and approximately 90% occurred in Sub Saharan Africa[1]. In the most heavily affected countries, such as South Africa, Botswana and Zimbabwe, HIV is the underlying reason for more than one third of all deaths among children under the age of five and this is reversing previous gains in child survival[1]. Prevention of mother to child transmission (PMTCT) interventions such as antiretroviral (ARV) prophylaxis have dramatically reduced the risk of vertical transmission from around 40% to less than 5% in some research and pilot settings in Sub Saharan Africa[2]. Governments have committed themselves to reduce the proportion of infants infected with HIV by 50 per cent by 2010, by ensuring that 80% of women have access to PMTCT interventions[3]. However recent data show that overall coverage of ARVs for HIV positive pregnant women is 33%[4] and there is poor coverage in countries with the greatest number of pregnant women living with HIV such as South Africa (50% coverage[5]), Nigeria (3% coverage[5]) and Tanzania (15% coverage[5]).

Programme evaluations from a number of countries in Africa have found deficiencies in various components of PMTCT programmes including uptake of antenatal HIV testing[6,7], receipt of test results[8], uptake of ARV prophylaxis [9-11] and postnatal mother-infant follow up[6]. South Africa has implemented a PMTCT programme since 2002. However, its impact has not been significant[12]. Whilst HIV testing had reached 69% in 2006/2007[13], a third of HIV positive women did not receive nevirapine[13] despite the country having an antenatal coverage rate of above 90%[14] and 84% of births assisted by trained health personnel[14].

Integrating PMTCT programmes into an already overburdened health system has been identified as a common reason for the sub-optimal performance of PMTCT programmes as they scale up to achieve national coverage. There is little documentation of how these challenges have been/are being addressed in routine (programmatic) settings where PMTCT programmes are taken to scale but evidence suggests that 'one size fits all' clinical guidelines are not leading to the desired changes[15]. This paper reports on the results of a participatory intervention to improve an integrated PMTCT programme in a rural district in South Africa.

## Methods

### Background

The package of care for the PMTCT programme at the time of this intervention included routine offer of antenatal voluntary counselling and testing (VCT), infant feeding counselling, single dose nevirapine to mothers and infants, infant PCR testing at six weeks and six months of free formula milk to women choosing not to breastfeed[16]. The PMTCT policy in the country has recently been revised to include dual short course prophylaxis consisting of AZT from 28 weeks together with single dose nevirapine to mothers and nevirapine plus seven days of AZT to infants[17].

### Setting

South Africa has a district health system in which comprehensive primary health care (PHC) clinics provide primary level care, referring patients to district and regional hospitals for secondary level care. PHC services are nurse driven. Clinic nurses are responsible for the diagnosis and management of infectious diseases such as tuberculosis, HIV and sexually transmitted infections, preventive care such as childhood immunisations and growth monitoring, antenatal care, as well as providing an acute curative service and attending to chronic conditions such as hypertension and diabetes.

The intervention was carried out in one district in KwaZulu-Natal province, Amajuba. In 2006 the district was estimated to have a total population of 585 858 and a population under one year of 13 259[14]. The antenatal HIV prevalence in 2006 was 46%, the highest in the country[18]. The district has a total of 3 hospitals, 18 comprehensive PHC clinics and 7 mobile clinics. The fixed clinics were included in the intervention but not the mobile clinics. PMTCT services are offered through comprehensive PHC clinics (antenatal HIV testing, CD4 count and provision of nevirapine) and the intrapartum component within the three district hospitals (delivery, provision of nevirapine if not already taken and administration of infant nevirapine syrup). All facilities have facility managers who together with the PHC supervisors and district programme co-ordinators represent the middle level of management in the health system.

Routine maternal and child health indicators for the district are good with an antenatal care coverage rate of 94%; 92% of deliveries undertaken by trained health professionals (midwives or doctors) and an immunisation coverage rate under one year of 83%[14]. PMTCT was introduced into the district in 2002 and whilst the uptake of HIV testing has increased from 30% in 2003/2004 to 78% in 2006/2007, other indicators have not shown much improvement. For example nevirapine coverage to women was 45% in 2003/2004 and 57% in 2006/2007 despite the programme being in its 5^th ^year[13]. Anthropological research in South Africa[19] has identified several health systems failures as contributing to the low uptake including non availability of counsellors and lack of testing supplies and consent forms.

### Intervention Design

The intervention consisted of a participatory assessment phase followed by a feedback and planning phase and then an implementation and monitoring phase. Each phase of the intervention had a focus on using routine data for problem identification, target setting and monitoring (Table 1).

**Table 1:** Description of the three intervention phases

**Participatory assessment phase**	
*Purpose: District programme managers gain skills in programme assessment*. *Formation of task team (programme managers and supervisors)*	
1. Training workshop on assessment framework and tools including piloting	
2. Formation of teams of 3-4 people	
3. Complete assessment of PHC facilities including interviews with facility managers, observation of facility functioning and interviews with lay counsellors.	
4. Collection of routine PMTCT data from district information officer.	
Description of tools:	
**Facility manager questionnaire**	*Structured interviews *with facility managers of PHC clinics. This tool assesses current staffing levels within clinics, organisation of services (days of the week and hours services are available), availability of lay counsellors, counselling services, laboratory services, supervision, health information, community involvement and roles and responsibilities of staff.
	
**Facility observations -- PHC clinics**	*An observational tool -- i.e. no interviews needed*. This tool assesses availability of drugs and supplies, rooms for counselling, documentation and record keeping.
	
**Counsellor questionnaire**	*Purposive sampling of at least half of the counsellors in each facility for a structured interview*. This tool assess training, relationships with clinic staff, space availability, waiting times, roles and responsibilities.
	
**Feedback and planning phase**	
*Purpose: District programme managers identify areas of weakness and learn to set realistic targets and action plans*	
1. Review of assessment results at a workshop.	
2. Identification of areas of weakness (e.g. infant PCR testing)	
3. Target setting and action plans	
**Implementation and monitoring phase**	
*Purpose: Team agrees on an action plan to address programme weaknesses. Continued support to sustain motivation and momentum*.	
1. Planned interventions implemented (road show to orientate staff to the PMTCT protocol and encourage joint responsibility amongst health workers)	
2. Monthly support visits by project facilitator (senior experienced professional nurse) to assess routine programme indicators and determine progress towards targets.	
3. Development of further action plans	

During the participatory assessment phase a task team consisting of programme managers for HIV, PMTCT, maternal and child health (MCH), unit managers for hospital labour and postnatal wards and PHC clinic supervisors was formed to improve the performance of the PMTCT programme. The purpose of the participatory assessment phase was to build the capacity of local programme managers to conduct a simple assessment of maternal and child health services in their district. The process was introduced at a workshop held with this team in May 2007. During the workshop the team was oriented to the assessment framework, introduced to the assessment tools and supported in a short phase of piloting. An evaluation guide was developed to give step by step instructions about how to plan, prepare for and undertake the assessments.

Three data collection tools were developed for the assessments; a structured interview tool for facility managers, an observation tool for PHC clinics and a structured interview tool for lay counsellors. These tools are described in Table 1. The conceptual framework chosen for development of the tools was based on an expanded health systems approach which has been proposed for evaluating PMTCT programmes[20]. This framework is based on the critical conditions managers need to consider in ensuring that a programme moves from efficacy (a programme's capacity to reduce the problem in ideal conditions) to effectiveness (its capacity to improve a problem in routine field conditions)[21]. The domains used in the assessment tools are: quality of services and human resources, availability of key resources and management systems and access and continued use of services.

Assessment teams consisted of three to four people (district and sub-district co ordinators and PHC supervisors) who visited facilities over a one week period. Each facility visit took approximately 3-4 hours. Assessment of the entire district took 7 days. Routine district PMTCT data from the district information officer was also collected in order to assess performance of key PMTCT indicators.

The feedback and planning phase and the intervention and monitoring phase are described in the results section of the paper as these were developed following review of the findings of the participatory assessments.

### Sampling

All eighteen comprehensive PHC clinics were visited in the assessment phase. At three facilities the facility manager was not present at the time of the assessment due to meetings or training resulting in a total of 15 facility manager interviews. Sampling of lay counsellors was determined by their availability but at least half of the total number assigned to each facility were included resulting in a total of 35 lay counsellor interviews.

### Data analysis

Interview and observation tools were submitted at the end of each day to the project facilitator who entered the data into excel. Epi-Info was used to generate basic frequencies for all tracer indicators as shown in Table 2. Routine district PMTCT data was extracted from the District Health Information System (DHIS) and analysed using excel. Routine PMTCT indicators were calculated for the six month period prior to the assessment. Basic data quality checks were done by the project facilitator and any errors identified with the indicators (for example, coverage levels over 100%) were verified with the district information officer. The three key conditions of effectiveness were used as the analysis framework.

**Table 2:** Key input and output indicators for Amajuba District collected during the participatory assessment phase

**KEY INPUT INDICATORS**		
**Condition of Effectiveness**	**Tracer indicator**	**n (Frequency)**

Quality of services and human resources	% clinical staff trained in PMTCT	28/105 (27%)
	% clinical staff trained in HIV and infant feeding counseling	29/105 (28%)
	% clinical staff trained in HIV counselling and testing	48/105 (46%)
	% lay counsellors with accredited training	34/35 (97%)

Access and continued use of services	% facilities taking first antenatal bookings every day of the week	10/15 (67%)
	% facilities with CD4 testing available	12/15 (80%)
	% facilities with PCR testing available	14/15 (93%)

Availability of key resources and management systems	% facilities receiving at least one visit by the district MCH supervisor in the previous six months	7/15 (47%)
	% facilities receiving at least one visit by the district PHC supervisor in the previous six months	12/15 (80%)
	% facilities receiving at least one visit by the district PMTCT supervisor in the previous six months	5/15 (33%)
	% facilities out of stock of rapid test kits	1/15 (7%)
	% facilities out of stock of nevirapine tablets	1/15 (7%)
	% facilities with IEC materials about VCT	11/15 (73%)
	% lay counsellors with dedicated counselling rooms	16/34 (47%)

**KEY OUTPUT INDICATORS (January to June 2007)**		

Proportion antenatal clients tested for HIV		5350/6064 (88%)

CD4 testing rate		1220/3071 (40%)

Nevirapine uptake rate among pregnant women with HIV		1750/3071 (57%)

Nevirapine uptake rate among babies born to women with HIV		452/3071 (15%)

HIV testing rate amongst HIV exposed infants at 6 weeks		740/3071 (24%)

## Results

### Findings from the facility assessments

#### Quality of services and human resources

Clinics had on average 7 full time clinical staff who were mainly nurses; only two clinics had doctors on site. Whilst there are no national norms for doctors in PHC it is expected that 10-15% of patients in clinics will be seen by a doctor and therefore clinics are expected to have a visiting doctor once a week. Coverage of PMTCT training was found to be sub-optimal with less than a third of clinical staff trained in PMTCT and HIV and infant feeding (Table 2). Interviews with 15 facility managers revealed that staff who had not been on the formal training courses did not feel that they could provide PMTCT services leaving the responsibility to a few trained staff. Coverage of training for lay counsellors was good with only one clinic not having all lay counsellors trained in the 10 day national counselling course.

The median number of clients seen per lay counsellor per day was 9 (range 2-30). This is slightly less than would be expected of a full time counsellor (10-12) but is dependent on the size of the facility and the number of nurses available to perform rapid HIV testing. Less than half of the lay counsellors reported having their own dedicated counselling room and 56% reported having to wait for a room to become available before they could counsel a client. 48% reported that this occurred on a daily basis.

#### Availability of key resources and management systems

Management of drugs and supplies was good with only one clinic found to be out of stock of rapid HIV test kits and one facility did not have nevirapine tablets. Documentation and record keeping was generally found to be good with all clinics having an HIV testing register and a nevirapine drug register which were up to date. All clinics had guidelines for HIV testing, however, a quarter did not have a PMTCT manual on site.

Supervision systems however were found to be poor. Forty seven percent of facilities had been visited by the district MCH supervisor in the previous six months and 33% had been visited by the district PMTCT supervisor in the previous six months. Supervision from the PHC clinic supervisors was better with a median of 3 visits per clinic (range 0-7) in the previous six months. These fall far short of the national norm of one visit per month to each facility.

#### Access and continued use of services

All of the clinics visited were open 5 days a week until 4 pm, however, a third of the clinics did not take first antenatal bookings every day of the week. Three clinics did not take blood for CD4 testing on site but referred clients to another facility. The South African PMTCT policy states that a CD4 cell count should be taken on the same day that the HIV positive status is established, and preferably at the first ANC visit therefore all clinics should be drawing blood for the CD4 test. The median turn around time for CD4 results was one week (range 1-5 weeks). This is better than the nationally recommended turn around time of two weeks. All of the clinics except one provided infant PCR testing and the median turn around time for PCR results was 6 weeks (range 1-24 weeks). National PMTCT guidelines state that all facilities should have the capacity to collect dried blood spots for PCR testing of infants. There is no South African norm for turn around time however WHO recommends a turn around time (from collection of sample to return of results) of no more than four weeks.

The review of routine PMTCT data for the period January to June 2007 (Table 2) showed that HIV testing was high at above 85%. CD4 testing was low with less than half of the HIV positive women receiving a CD4 test. Slightly over half of HIV positive women received nevirapine and only 15% of infants receiving nevirapine. Only a quarter of infants received a PCR test at 6 weeks.

### Development of the quality improvement intervention

The results of the assessment were reviewed during a workshop with the assessment team and key district managers. The participants were divided into groups according to key areas of weakness identified during the assessment. The teams were asked to think through the reasons for these weaknesses. The main reasons given by the groups are presented in Table 3. The number of health workers who felt that they did not have responsibility for PMTCT because of a lack of formal training was particularly highlighted as a cause of poor coverage. In response to these results the team laid out a plan of action for addressing key areas of weakness. These included improving the knowledge and orientation of all levels of clinical staff about PMTCT and using a data driven approach to increasing awareness within the district of the current performance for key PMTCT indicators in order to encourage joint responsibility for improving coverage.

**Table 3:** Responses to weak areas identified during the assessment from group work undertaken with the assessment teams and key district management.

**Reasons for the low coverage of nevirapine to women and infants**	**Reasons for the low coverage of CD4 testing amongst HIV positive women**	**Reasons for the low coverage of PCR testing amongst HIV exposed infants at 6 weeks**
**Client factors:** clients not clear about when to take nevirapine. unbooked patients/late booking **Health system factors:** lack of training of nursing staff lack of privacy in labour wards thus difficult to ask about HIV status nurses not checking HIV status on medical records lack of ownership of the PMTCT programme by staff poor recording of nevirapine doses during ANC/labour. nevirapine not available or locked in a cupboard	**Health system factors:** shortage of staff unclear roles & responsibilities poor recording of HIV status on antenatal records some facilities only 1 person in charge of HIV and responsible for all CD4 testing lack of staff knowledge and motivation lack of clarity on the PMTCT protocol low confidence in interpretation of policies/protocols	**Client factors:** mothers fear of disclosing status or of knowing child's status infants brought to clinic by nanny or grandmothers which results in consent issues for testing mothers not being adequately informed **Health system factors:** no tracing system for defaulters staff shortages whose role is it? registered nurse/enrolled nurse too few staff trained to do PCR -- seen as complicated procedure coding system on RTHC difficult to understand poor/no recording on RTHC

The team developed a district wide 'road show' which consisted of a half-day orientation package that could be delivered at each facility. The package consisted of an overview of the PMTCT protocol and how it fits within MCH services, a comparison of key PMTCT indicators between facilities to enable staff to assess their own performance and to discuss areas for improvement followed by the development of action plans and targets.

A process of regular monthly support for facilities was undertaken by a project funded district facilitator together with the district supervisors. During these visits a data driven approach was used in assessing the previous month's indicators and determining progress towards targets. At each of these visits further action plans were developed.

### Early effects of the intervention

**Figure 1:**
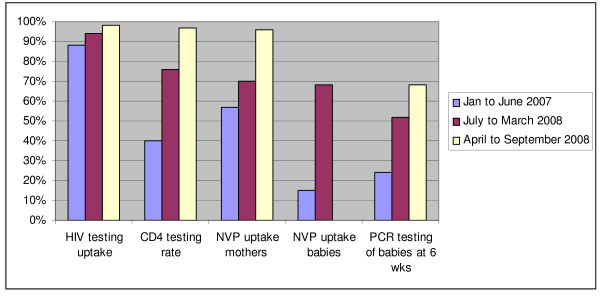
PMTCT Indicators for Amajuba District

Routine PMTCT indicators were monitored monthly for a year following the intervention. Improvements were noted in all key PMTCT output indicators (Figure 1). Antenatal HIV testing although high prior to the intervention, increased to 98%, CD4 testing of HIV positive mothers increased from 40 to 97%, maternal nevirapine from 57 to 96% and infant nevirapine from 15 to 68%.

An increase in 6 week PCR testing from 24 to 68% was achieved through improved identification of HIV exposed infants. Clinics implemented accelerated efforts to identify HIV exposed infants through asking mothers about their HIV status and improving recording of infant exposure status on the routine child health cards. Facility managers assessed facility registers on a weekly basis to determine whether the expected number of infants attending 6 week immunisation visits (approximately 46% based on the district antenatal HIV prevalence) were receiving a PCR test.

Assuming 4837 births to HIV positive women per year in the district[13] and 12% perinatal transmission with 100% coverage of the PMTCT regimen[22] it is estimated that the improvements in coverage will have averted approximately 580 infant HIV infections per year.

## Discussion

A common complaint amongst programme managers is the large amount of data they have to collect and process. The development of a comprehensive health systems framework, key tracer indicators and relatively simple data collection tools in this intervention enabled middle level managers to feasibly collect relevant data rapidly and to identify possible bottle necks to optimal programme performance.

The results of the assessment showed that the district is clearly performing well in terms of HIV testing within antenatal care and this has been achieved through adequate human resource provision in terms of lay counsellors and a philosophy of making HIV testing a routine component of antenatal care. Areas of weakness identified include inadequate coverage of training amongst clinical staff, inadequate infrastructure in terms of counselling rooms, infrequent supervision by district supervisors, low coverage of CD4 testing, nevirapine to mother and infant and infant PCR testing. These weaknesses are due to the complex interaction of client and health systems factors. Client factors include lack of information and fear of disclosing HIV status, health systems factors include lack of ownership of the PMTCT programme amongst nurses, unclear roles and responsibilities, lack of knowledge of the protocol, poor recording systems and continuity of care.

The process of identifying weak areas and implementation of a participatory approach to problem solving together with regular, data driven, facility level support resulted in large increases in key programme indicators over the period of one year.

An intervention in Zimbabwe[10] to improve PMTCT using a district approach including regular team meetings was also found to be successful in terms of HIV test acceptance. Continuous quality improvement interventions using a data driven approach have been advocated to ensure sustainable local improvements in health programmes [23,24]. These types of interventions use several quality improvement cycles to identify problem areas, using local programme data to set targets and for continuous monitoring throughout the process. Quality improvement studies in developed countries have found that feedback on performance and small group meetings have shown positive effects on changing health worker behaviour[25]. One of only two reviews including studies from low income countries has also suggested that approaches such as supportive supervision and audit with feedback may be effective in improving health worker performance in these settings[26].

This intervention used a participatory approach which has both advantages and disadvantages. The disadvantages are that it relies on support and buy in from senior district management to allow mid level managers the time to participate in the workshops and to actually undertake the assessments. Without this support, the participatory approach would not succeed. It is also possible that there is some loss of data quality by utilising health workers to collect assessment data instead of trained research data collectors however the aim of the project was not to be a rigorous research study but rather to develop the skills of mid level managers to conduct assessments of their own programmes and to interpret and act on the findings. The advantages of a participatory approach are that the process of conducting the assessments enables mid level managers to see first hand how well their facilities are functioning and to take ownership of the findings since it is data that they themselves have collected.

This operational research study has several limitations which deserve discussion. Firstly the assessment of the intervention relied on routinely collected primary health care data. Routine health system data for PMTCT in South Africa has been found to suffer from problems of completeness and accuracy[27] and this was taken into account during the intervention. Since a focus of the intervention was on using data to assess programme performance, the project facilitator helped supervisors to identify data errors during monthly facility visits and attempts were made to correct the data as much as possible, however some minor errors may still exist.

Secondly the operational research design did not include a control or comparison group hence the findings cannot be causally attributed to the intervention. However, the intervention was adopted and supported by the district management team as something that would be conducted across the entire district and during its implementation there were no other large scale efforts by NGOs or the district health department to address the quality of the PMTCT programme. The changes reported here were observed over a period of approximately 18 months prior to the introduction of the new PMTCT regimen when no media activities or health system changes were occurring. It is therefore plausible that the effects seen are due to this intervention[28].

Improving PMTCT programmes is vital if the worsening under five mortality in high HIV burden countries such as South Africa is to be reversed. This is unlikely to be achieved through resource intensive generic health-system interventions (e.g. training courses and development of protocols and guidelines). The impact of these, in terms of programme functioning and health outcomes, has been disappointing; this has been attributed to their failure to link with specific programme activities[29]. We propose that a more participatory operational research approach that encourages a partnership between managers and researchers, such as outlined in this paper, will be more successful in improving the quality of PMTCT programmes. Furthermore, to ensure sustainability of improvements, it is recommended that ongoing support and supervision is needed from an experienced facilitator in order to establish a culture of data driven monitoring and to foster greater ownership of programme performance.

This study was undertaken in one district with a relatively well functioning basic PHC system. The findings therefore cannot be generalised to every district in South Africa. All districts in the country do however have similar district management structures that would be applicable to this form of intervention. The addition of a facilitator for the duration of the intervention does add to the cost however it has been found to be essential at least in the initial stages of the intervention to encourage motivation and focus on the improvement targets. This approach of providing ongoing support through an external facilitator has recently been adopted as part of a national government accelerated plan for PMTCT. Several large NGOs have entered into performance contracts with the government which includes provision of external support for quality improvement.

## Conclusion

The development of a comprehensive programme evaluation framework, identification of key indicators and relatively simple data collection tools in this intervention enabled district and sub-district level managers to collect relevant data rapidly on the individual effectiveness of each programme component, as well as identifying possible efficiency gains and missed opportunities for improved programme performance. This approach could be scaled up as a model for participatory programme improvement.

## Competing interests

The authors declare that they have no competing interests.

## Authors' contributions

TD, MC and DN participated in the design of the study, conducted training and drafted the manuscript. TD and DN analysed the data. DM participated in the coordination and implementation of the study and helped to draft the manuscript. All authors read and approved the final manuscript.

## Pre-publication history

The pre-publication history for this paper can be accessed here:


